# Intravascular Application of Labelled Cell Spheroids: An Approach for Ischemic Peripheral Artery Disease

**DOI:** 10.3390/ijms22136831

**Published:** 2021-06-25

**Authors:** Jörg Schmehl, Hartmut Stoll, Marina Danalache, Gerd Christian Grözinger, Tim-Oliver Greiner, Rebecca Felizitas Leibfritz, Petros Martirosian, Konstantin Nikolaou, Stefanie Elser

**Affiliations:** 1 Department of Diagnostic and Interventional Radiology, University Hospital of Tübingen, D-72076 Tübingen, Germany; joerg.schmehl@med.uni-tuebingen.de (J.S.); hartmut.stoll@med.uni-tuebingen.de (H.S.); gerd.groezinger@med.uni-tuebingen.de (G.C.G.); Rebecca.Leibfritz@med.uni-tuebingen.de (R.F.L.); petros.martirosian@med.uni-tuebingen.de (P.M.); konstantin.nikolaou@med.uni-tuebingen.de (K.N.); stefanie.elser@med.uni-tuebingen.de (S.E.); 2 Department of Orthopedic Surgery, University Hospital of Tübingen, D-72076 Tübingen, Germany; 3 Department of Experimental Surgery, University Hospital of Tübingen, D-72076 Tübingen, Germany; vet@exp-med.de

**Keywords:** 3D spheroids, regenerative medicine, magnetic resonance imaging, peripheral artery disease, critical limb ischemia, mesenchymal stem cells, endovascular application, small particles of iron oxide

## Abstract

Mesenchymal stem cells (MSC) are known for their vascular regeneration capacity by neoangiogenesis. Even though, several delivery approaches exist, particularly in the case of intravascular delivery, only limited number of cells reach the targeted tissue and are not able to remain on site. Applicated cells exhibit poor survival accompanied with a loss of functionality. Moreover, cell application techniques lead to cell death and impede the overall MSC function and survival. 3D cell spheroids mimic the physiological microenvironment, thus, overcoming these limitations. Therefore, in this study we aimed to evaluate and assess the feasibility of 3D MSCs spheroids for endovascular application, for treatment of ischemic peripheral vascular pathologies. Multicellular 3D MSC spheroids were generated at different cell seeding densities, labelled with ultra-small particles of iron oxide (USPIO) and investigated in vitro in terms of morphology, size distribution, mechanical stability as well as ex vivo with magnetic resonance imaging (MRI) to assess their trackability and distribution. Generated 3D spheroids were stable, viable, maintained stem cell phenotype and were easily trackable and visualized via MRI. MSC 3D spheroids are suitable candidates for endovascular delivery approaches in the context of ischemic peripheral vascular pathologies.

## 1. Introduction

Peripheral arterial disease (PAD) is characterized by reduction of blood perfusion in tissues of the lower extremities [1]. The prevalence of critical limb ischemia (CLI), an advanced stage of PAD is markedly increasing, accompanied by a high mortality rate [2]. CLI prevalence increases with age and comorbidities like diabetes mellitus [3]. Patients suffering from CLI represent a challenging population to be treated medically, surgically, and endovascularly [2].

The symptoms of the disease differ depending on the localization and size of the vessels affected. Even though, therapeutic options like endovascular therapy or bypass surgery exist for the crural arteries, affected smaller peripheral vessels are usually not accessible by such mechanical approaches [4]. In this context, the use of stem cell—based therapies have been proposed as a suitable therapeutic option [5,6]. The aim of cell-based therapies is to restore tissue perfusion and thereby promote wound healing in CLI [7]. Mesenchymal stem cells (MSC) [8,9] and endothelial progenitor cells (ECFC) [10,11] are preferred as cellular components in the field of therapeutic angiogenesis, particularly due to their direct formation of new vascular structures and immune—modulatory properties. The clinical efficacy of MSCs is based on their multipotent differentiation capacity towards vascular smooth muscle cells, endothelial cells and other cell types, as well as their ability to secrete trophic factors. These factors are known to promote angiogenesis, thus, inhibiting apoptosis and modulating immunoreactions [5]. Moreover, early phase clinical trials have shown that stem cell therapies are feasible as well as safe and effective [12]. However, the need for substantial preparation and expansion of the cell therapeutics limits their routine use. There is an unmet need for new cell application procedures that are reasonably balanced in terms of costs and clinical benefit. 

Current experimental and clinical research on MSC vascular therapies generally utilizes local administration of cells. Local delivery directly targets cells in the injured region. In fact, local application of MSCs has already been investigated in the context of peripheral arterial pathology, particularly in the case of diabetes neovascularization [13].

A key requirement for a successful therapeutic approach with MSCs is an adequate number of vital and functional cells that need to be delivered in the targeted region of tissue damage, for a direct regenerative effect. In clinical trials, the predominant delivery method is intramuscular injection, yet this approach often cannot deliver cells in the targeted region. Another approach is represented by intra-arterial injections; however, it does not result in sufficient local cell concentrations, due to systemic distribution of cells in other organs [14,15,16]. An approach to address this limitation and increase the number of cells in the affected tissue, could be the use of 3D cellular spheroids. Compared with the routinely used 2D monolayer culture, 3D spheroidal cell aggregates represent a more physiological approach, with a wider range of application fields: oncology, stem cell biology and tissue engineering [17,18]. Furthermore, as reported in previous trials [7], injected 2D cultured cells showed a poor survival in the ischemic environment with impaired function. 

MSC—spheroids consist of a 3D- microstructure with a core of densely packed cells with an outer monolayer of cells, cells on the periphery of the spheroid proliferate more actively [19]. Moreover, 3D cultivated MSCs show an improved cell survival after delivery and are characterized by enhanced anti-inflammatory, angiogenic and regenerative capabilities [17].

Another key aspect towards a clinical translation of cell therapy approaches is cell tracking. The current available approaches for cell tracking include imaging with MRI, positron emission tomography, single photon emission computed tomography, and fluorescence imaging [20]. In this context, MRI is a routinely used imaging modality for cell tracking that enables noninvasively the visualization of labelled transplanted cells. As contrast agents superparamagnetic (SPIO) or ultrasmall superparamagnetic iron oxide particles (USPIO) are used [20]. The latter being known as harmless and non-cytotoxic, thus, unaltering cellular viability, proliferation, and differentiation both in vivo and in vitro [21,22]. 

In particular, patients suffering from circulatory disorders with impaired micro perfusion and restricted mechanical access to the affected vessels can benefit from the spheroidal application approach to avoid major amputation. Therefore, in the present study we evaluated the feasibility of intravascular application of labelled 3D MSC spheroids by assessing the influence of the application procedure on viability, integrity and functionality and the behavior in an ex vivo muscle injection model. 

## 2. Results

### 2.1. Size Distribution of 3D Cell Spheroids

**Figure 1:**
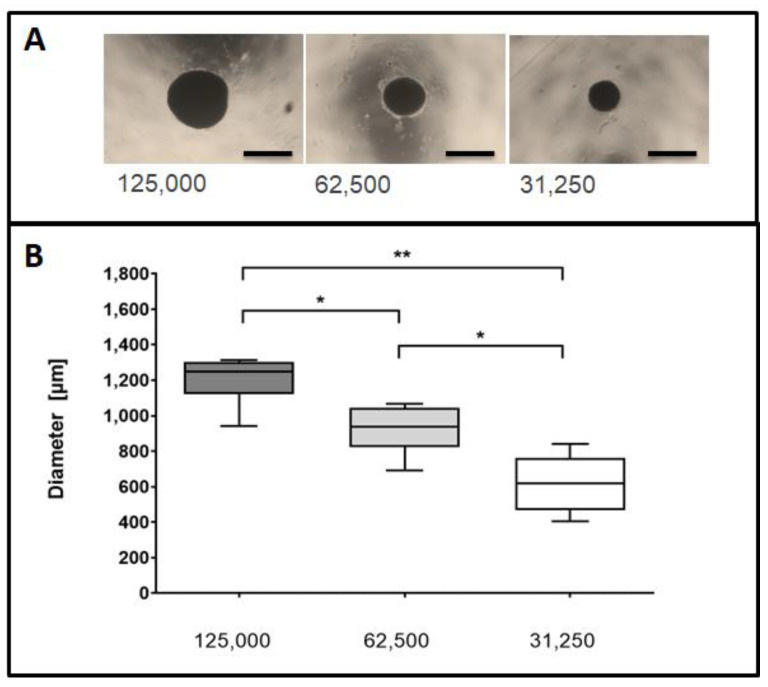
Assessment of spheroidal diameters at different seeding densities. (**A**) Representative images of spheroids using different initial seeding densities. Cell spheroids were seeded at densities of 125,000, 62,500 or 31,250 cells and visualized by phase contrast microscopy 5 days post seeding. Size bars (black) represent 1000 µm at a 2.5× magnification. (**B**) Spheroidal cross-sectional areas were measured for the imaged spheroids. Data are taken from six independent experiments and depicted in a Box and Whisker Plot. Differences between groups were analyzed by a Mann-Whitney test. * *p* < 0.05, ** *p* < 0.01.

The MSC spheroids were generated with three different cell densities (125,000; 62,500 and respectively 31,250 cells) and formed by self-aggregation, without the addition of exogenous extra-cellular matrix components (ECM). MSC cells formed spheroids with a spherical, regular and compact shape (Figure 1A).

The measurements of the MSC cell spheroids yielded a mean diameter of 1205.6 ± 43 µm for spheroids composed of 125,000 cells, 923.1 ± 35 µm in case of 62,500 cells and 618.9 ± 24 µm for 31,250 cells (Figure 1B, *n* = 6 independent experiments).

### 2.2. Phase Contrast Microscopy and Scanning Electron Microscopy (SEM) Imaging of MSC Spheroids

**Figure 2:**
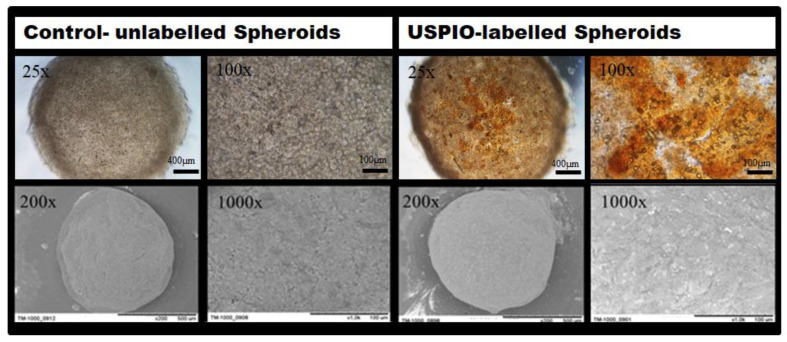
Phase contrast images and SEM images of native (**left column**) and USPIO-labelled MSC spheroids (**right column**) with an initial cell density of 125,000 cells. Upper row: Phase contrast imaging of spheroids. The distribution of USPIOs appears as brown-colored areas (25× and 100× magnification). Bottom row: SEM imaging of spheroidal surfaces. Pictures show the surface structure of 5-days old spheroids (200×, and 1000× magnification).

To confirm USPIO-labelling of the spheroids, the labelled cells were collected, seeded into spheroidal well plates and visualized via phase contrast microscopy after 4 h (*n* = 3). Labelling of the cells was demonstrated by the presence of brown colouring of the spheroids due to the Ferucarbotran USPIO particles. An irregular distribution of the USPIOs was detected (Figure 2—upper row).

For assessment of micro-structural characteristics of USPIO-labelled spheroids, scanning electron microscopy (SEM) was carried out. Minor structural modifications at the surface of the labelled spheroids caused by the Ferucarbotran particles were noted (Figure 2, bottom row). The spheroids’ surface appeared in grained texture and exhibited a light silver-white sheen when compared to untreated cell spheroids. Cellular labelling did not, however, affect the formation of spheroids with a characteristic spherical shape (Figure 2, bottom row). It is noteworthy that the iron clusters were detected extracellularly for the USPIO-labelled spheroids.

### 2.3. Mechanical and Cell Physiological Assessment of Spheroids after Application

**Figure 3:**
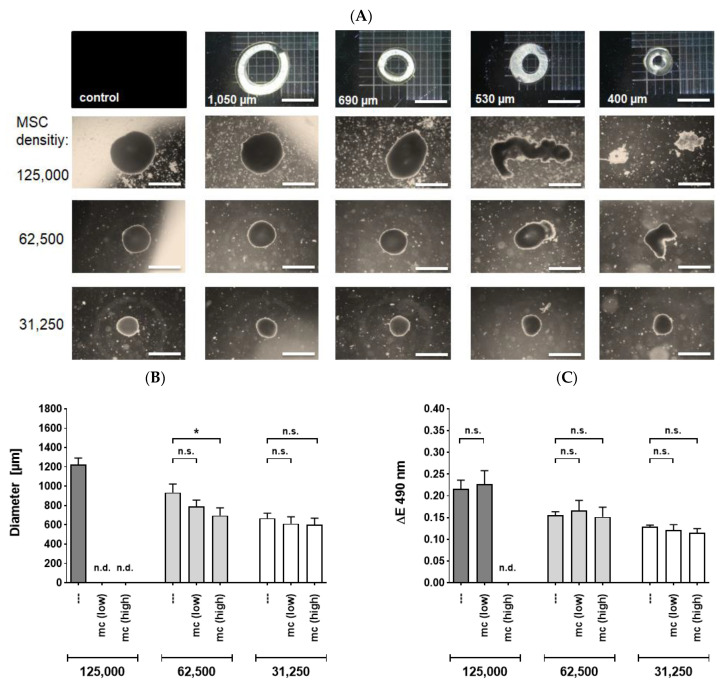
MSC spheroids applied through capillary tubes or microcatheters with various sizes. (**A**) Phase contrast microscopy (2.5× magnification, size bar 1000 µm) of spheroidal integrity after application through capillary tubes. Spheroids seeded at different initial cell densities were applied through flexible tubes with inner diameters of 1050 µm, 690 µm, 530 µm or 400 µm, respectively. Spheroids were partially deformed or disrupted at critical tube diameters. (**B**,**C**) Deformation and viability of spheroids after application with different flow rates through a microcatheter: Spheroids were applied with standard (low, 90 µL/s) or high (200 µL/s) flow rates through a 530 µm sized microcatheter and collected in well plates. Subsequently, diameters of spheroids were measured (**B**) or spheroidal viability was determined using MTS assay (**C**). Cell spheroids of 125,000 MSCs which were slightly disrupted at low velocity were used for viability assessment but not for size determination. Data from four to six independent experiments is depicted. Bars represent means ± Standard Error of the Mean. Differences were analyzed by a Mann-Whitney test. * *p* < 0.05; Abbreviations: mc—microcatheter; n.d.—not determinable; n.s.—not significant.

We investigated the resilience of MSC spheroids of various sizes, applied through flexible tubes of a capillary phantom to assess the deformation effects. We investigated the overall cellular survival of the 3D spheroids after the application procedure through different catheter lumen sizes. When spheroids were applied with a standard velocity of approximately 90 µL/s, different effects on their structural integrity were noted in accordance to the size of the spheroids and the inner diameter of the tubes (Figure 3A, *n* = 4–6). At higher ratios of spheroidal sizes to inner lumen diameters, an increasing deformation of the spheroids was observed, resulting in an elongated, cylindrical shape of the spheroids. A notable morphological disruption occurred at spheroid to inner lumen diameter ratios of 2.3 and higher (Figure 3A) followed by a complete loss of coherent structure and formation of numerous fragments at ratios above 2.8. Spheroids retained their full integrity or were only slightly deformed, when applied through tubes with inner diameters at least half as large as those of the spheroids. We observed that a ratio of 2.2 represents a threshold for an integer coherent structure of MSC spheroids under standard conditions.

The velocity rate of 90 µL/s represented an adequate and mild condition in terms of physical pressure. However, in a clinical setting the spheroid application might be subjected to variable degrees of mechanical stress. We therefore addressed the question whether the spheroids’ integrity and viability are preserved under more stringent conditions. For this purpose, we used a microcatheter with a lumen size of 530 µm, which is routinely used in a clinical context of peripheral artery pathologies.

Applied 62,500-MSC spheroids yielded a sufficient number of intact, cohesive spheroidal structures for both high and low velocities. Cell spheroids consisting of 62,500 were deformed significantly at a high velocity but not at low rates (Figure 3B, *n* = 4–6). However, the viability of the MSC cells was not affected at none of the flow rates employed (Figure 3C, *n* = 4–6). Additionally, no significant effects in terms of deformation or viability were noted for 32,500 cell spheroids (Figure 3A–C). The inner lumen size of the utilized capillary tubes or catheters was a decisive factor for the structural integrity and viability of the MSC spheroids. An elevated velocity rate had only minor effects.

**Figure 4:**
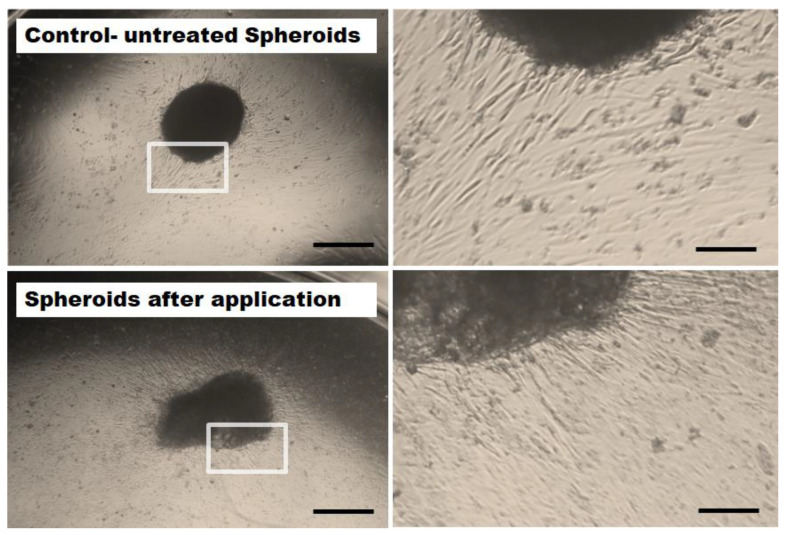
Representative microscopic images of MSC outgrow potential. 62,500 MSC spheroids (5 days old) were retrieved after application with high velocity through a microcatheter (inner diameter 530 µm, bottom row) and cultivated in well plates. As control, unapplied spheroids were employed (upper row). Irrespective of the flow rates, cells from all intact or slightly deformed spheroids spread out evenly and attached to the surface of the cell culture wells. Images were taken with a 2.5× magnification (left column). Size bars indicate 500 µm. The right column shows magnified details of the spheroids (enlarged 4-fold, size bars represent 125 µm).

Outgrowth of MSCs (originated from the applied spheroids) was investigated for all tested cell densities of spheroids (*n* = 6). After the application procedure, all of the intact, deformed or slightly disrupted spheroids maintained their capability to attach and showed an outgrowth of cells. The outgrowing cells had a spindle-type cell shape characteristic for MSCs. No microscopic difference in cellular outgrowth potential was observed at the different flow rates, indicating that high velocity rates did not crucially impair the outgrowth potential of MSCs (Figure 4).

### 2.4. Differentiation Potential of Applied and Untreated MSCs

**Figure 5:**
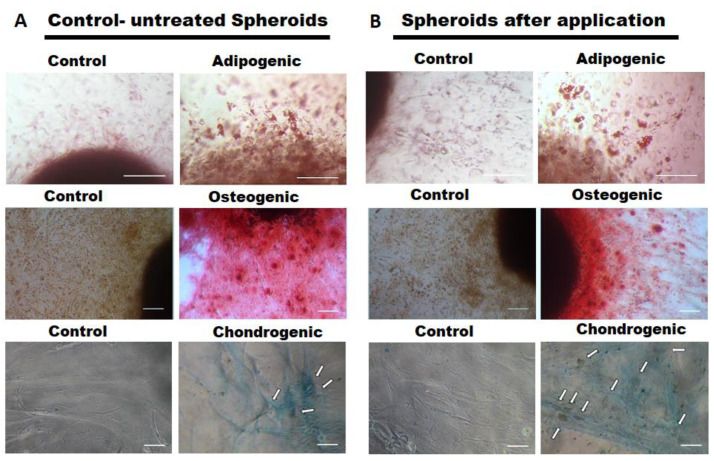
Differentiation potential of MSCs from spheroids injected through a microcatheter. Spheroids consisting of 62,500 MSCs were applied through a microcatheter (inner diameter 530 µm). As controls, unapplied MSCs were used (left columns). Differentiation capacity of the cells is visualized in the right columns. (**A**,**B**) upper row: adipogenic differentiated cells containing lipid vacuoles as determined by Oil Red staining (20× magnification, size bars 100 µm); (**A**,**B**) middle row: calcium deposits of osteogenic differentiated MSCs, visualized by Alizarin Red staining (10× magnification, size bars 100 µm); (**A**,**B**) bottom row: cells exhibiting a chondrocyte-like cell shape ingrained in a proteoglycan rich extracellular matrix as determined by Alcian Blue staining (white arrows indicate chondrocytic cell aggregates, 10× magnification, size bars 100 µm).

The differentiation potential into different lineages (adipogenic, osteogenic, chondrogenic) of microcatheter-treated and untreated control MSC spheroids was analyzed (*n* = 4–6). No notable difference was observed for the applied MSC spheroids (Figure 5B) when compared to the untreated controls (Figure 5A). Adipogenic differentiated cells containing lipid vacuoles were released from both applied and untreated differentiated spheroids to a similar extend (Figure 5A,B, upper row). Calcium deposits produced by osteogenic differentiated cells derived from both spheroid types indicated a comparable osteogenic differentiation potential (Figure 5A,B, middle row). Moreover, cells outgrown from both applied and untreated control spheroids similarly exhibited aggregations of chondrocytes embedded in a proteoglycan-rich extracellular matrix (Figure 5A,B, bottom row).

### 2.5. MR-Imaging of USPIO- Labelled Spheroids in an Agar Phantom and an Ex-Vivo Rabbit Hind Limb Model

**Figure 6:**
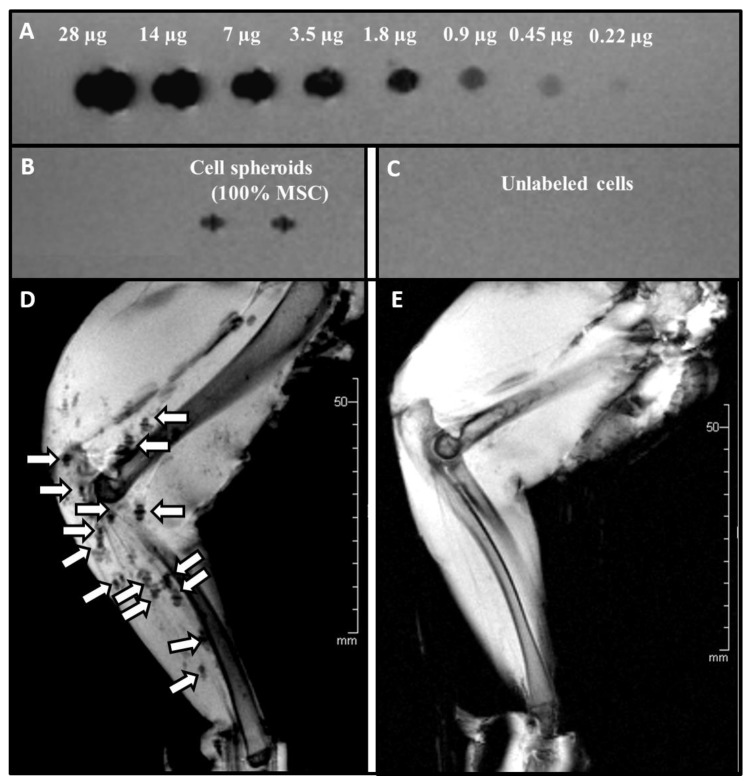
MRI Imaging of an Agar phantom and USPIO labelled/unlabelled MSC spheroids applied intra-arterial into a rabbit hind limb (**A**) Upper row: Descending concentration levels of Ferucarbotran (28µg to 0.22µg). (**B**) USPIO-labelled regenerative Cell spheroids consisting of 125,000 MSCs, and (**C**) 2 native cell spheroids without USPIO labelling (**D**) After the application procedure the distribution of the USPIO- labelled spheroids was visualized in the vessel bed/vasculature by a T2s-weighted MRI sequence. Unlabelled spheroids were used as control (**E**).

In order to validate the detectability and traceability of USPIO-labelled spheroids ex vivo, an agar phantom setting coupled with MRI imaging was employed (Figure 6, *n* = 3 independent experiments). Loading of decreasing concentration of USPIO particles in an agar phantom was clearly visualized in MRI up to a minimum load of 0.22 µg Ferucarbotran (Figure 6A). A similar signal was achieved by USPIO- labelled (60 µg/mL) 3D cell spheroids at a cellular density of 125,000 MSCs (Figure 6B). For unlabelled/control cells no signal was detected (Figure 6C).

Cell spheroids labelled with USPIO-containing media for 4 h exhibited no difference in the configuration. Application into the arterial system of freshly harvested muscular tissue showed an accumulation of the spheroids in small arteries with evenly distribution within the targeted vessels. The MRI images (Figure 6D,E) showed a homogenous dispersion of the labelled spheroids throughout the vascular territory (Figure 6D). In contrast, unlabelled cell spheroids could not be visualized (Figure 6E).

### 2.6. Histological Ex-Vivo Analysis of Applied USPIO-Labelled Spheroids

**Figure 7:**
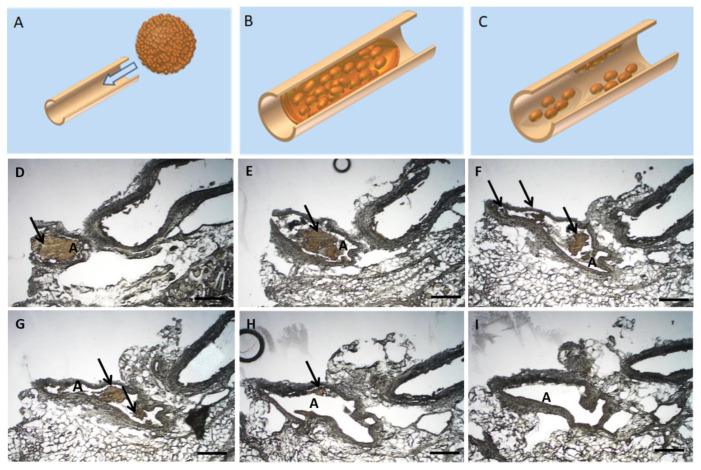
Histological ex-vivo analysis of applied USPIO-labelled spheroids. (**A**–**C**) Proof of concept: Schematical representation of spheroid passage through a rabbit muscle artery. Once the spheroid is applied through the artery (**A**), it elongates in shape to mimic the artery (**B**) and thus in time the cells migrate and spread throughout the affected vessel (**C**). Figure 7(**D**–**I**) Sequential histological specimen of vascular cross sections from a New Zealand Rabbit model. The regenerative cell spheroids were administered intra-arterially in the hind leg musculature. The USPIO-labelled MSC cells appear brown as a result of the USPIO labelling. Scalebar = 500 µm, A: Artery, Arrows indicate the USPIO-labelled spheroids.

Following the ex vivo application and MRI visualization, a histological assessment of USPIO-labelled spheroids (Resovist^®^/Ferucarbotran) was employed (Figure 7, *n* = 3). After injection into the femoral artery of a rabbit hind limb, the spheroids distribution was examined histologically. The spheroids spread evenly in peripheral and intramuscular vessels. As illustrated in the proof of concept, once applied the spheroids elongate thus mimicking the shape and the diameter of the vessels (Figure 7A,B). A disintegration and migration of cell aggregates occurred; thus, the targeted regenerative cells can reach the injured/affected region (Figure 7C). A similar behaviour of applied USPIO-labelled spheroids was observed in our sequential, histological assessment of applied USPIO-labelled spheroids. The MSC spheroids were easily identified histologically (Figure 7D) with a resulting fragmentation of the spheroids (Figure 7E–G) and migration of the cellular aggregates (Figure 7H,I).

## 3. Discussion

Compared to traditional 2D cell cultures, MSC spheroids are characterized by enhanced regenerative capacities combined with an upregulated secretion and production of trophic, anti-apoptotic and anti-inflammatory factors [23,24,25,26,27]. Only about 5% of injected single cell suspension cells persist at the site of injection within days after transplantation [28]. There is a need of effective methods to augment the distribution of spheroids as well as cell viability and functionality [29]. Cell based therapies for peripheral vascular disease have been investigated as an alternative for standard revascularization therapies like angioplasty or bypass grafting, when these methods fail [30]. In this study, we aimed to investigate the suitability and applicability for clinical translation of 3D MSCs spheroids in the context of peripheral artery pathologies. Particularly, the size reproducibility, deformation effects, viability, functionality as well as ex vivo tracking of USPIO labelled 3D spheroid distribution and disposition in small muscular arterioles were investigated.

By altering the initial cell density, we were able to generate 3D spheroids of various sizes in a reproductible manner. High cell densities were employed to achieve an increased spheroidal diameter size. These spheroids were prerequisite in order to obtain a notable deformation effect after application via clinical used catheters. Our data is in accordance with previous studies, where the formed spheroids were easily imaged optically due to their centered position and were easy to handle in further analysis [31,32]. Even tough, from a clinical perspective, a substantial cell density is required for cell-based therapies to trigger tissue formation, the increasing length of nutrient transport by diffusion arises as a limitation. While Schmitz et al. showed that MSC spheroids with diameters greater than 200 µm are vulnerable to hypoxia and implicitly cell death [33] several other studies investigated the presence of a hypoxic core by means of histological approaches and indicated the presence of proliferating cells in spheroids up to 1000 µm in diameter [29,34,35]. It has to be borne in mind that in a clinical context, for peripheral arterial disease, the spheroidal size has to be subjected to further evaluation. Particularly the total number of applied cells, in terms of either smaller and multiple spheroids vs. lower number of spheroids of higher diameters has to be further investigated. The presence of intra-spheroidal hypoxia might result in a priming of the MSCs for the subsequent application to ischemic tissue. Such, it facilitates the cellular adaptation process to an ischemic environment. In fact, intramuscularly applied spheroids in a hind limb, yielded an overall improved survival of translated cells, and increased number of de novo micro vessels [36]. To validate the efficiency of the application procedure, it is important to monitor the distribution and migration capabilities of the transplanted cells. We demonstrated that simple co-incubation was sufficient for labelling MSC spheroids with USPIOs, which were subsequently detected and tracked by MRI. While no morphological changes were observed in none of the spheroid groups, the USPIO labelled spheroids exhibited silver-white coating. In line with our results, Schäfer et al. also showed that for USPIO labelled cells the iron particles had an intracellularly and extracellularly localization, being associated predominantly with the exterior of the cell membrane [37]. Cellular uptake occurs through transferrin– or clathrin-mediated pathways [37,38] and is orchestrated by the physicochemical properties of targeted nanoparticles [39]. It has been assumed, that metabolism of USPIO particles occurs via lysosomal degradation mechanisms and iron released from endosomes is scavenged intracellularly by ferritin [40,41,42].

The ability to maintain integrity after injection is a key factor for the clinical translation of MSC spheroids. In this context we investigated the cellular function of MSC spheroids after their transit through various sizes of tube lumens, at two different flow rates (90 µL/s and 200 µL/s), the former comparable to those used in clinical studies. High flow rates and their corresponding wall shear stress have to be considered, as they do not only affect the cellular function by upregulating the secretion of pro-inflammatory cytokines [43] but also, the cellular viability [44,45]. The results of this study showed, that neither the cellular viability as assessed by MTS assay nor the outgrow features assessed microscopically show any significant changes with either flow rate of 90 µL/s or 200 µL/s. Heng et al. showed that when MSC were administrated by direct intramyocardial injection through a transfemoral 26G needle at flow rates similar to the ones used in our experiments (400 and 1600 µL/min), the gene expression of cytokines and growth factors (bFGF, SDF-1, SCF, VEGF) were significantly different at both flow rates, however, with no differences in the amounts of secreted proteins [44]. Shear forces encountered by MSCs during application have a minimal and transitory effect upon MSC molecular marker expression [44]. Notably, Walker et al. showed that applying single cell suspensions of human MSCs through 25G needles (similar in terms of size to our microcatheter at a comparable [46] flow rate of 140 µL/s) a significant decrease of viability after 24 h was noted [47]. In contrast, our results showed that 16 h post-application the MSC spheroids had an unaltered viability (no significant change of viability). Thus, it is conceivable that spheroids are capable to withstand the stress of injection into sites of interest due to their firm intercellular connections allowing a continuous cellular adaptation. An interesting observation of this study is that irrespective of the flow rate employed, the inner lumen size of the catheters used for application was a decisive factor in terms of the structural integrity and viability of the spheroids. It has been previously shown that smaller needle bore size increases apoptosis of ejected cells [48] which can be countered by slower flow rates [49]. Several publications (both on MSCs and other cell types) have indicated that cells found on the periphery of the spheroids had a high proliferative potential, while 80% of the cells from the spheroidal core zone remain in a cellular arrest in G0/G1 phase [24,29,46,50,51]. The spheroids were considered as transport vehicles, to yield a high number of regenerative and viable cells to an ischemic region. Even though, the spheroids are characterized by an intra-spheroidal hypoxic core, at higher ratios of spheroidal sizes to inner lumen catheter diameters, the applied spheroids are likely to undergo a reshaping process. Hence, probably exposing the central core cells and reactivating these cells from the quiescent phase.

The ability to differentiate and produce important cell lineage, such as osteocytes, chondrocytes and adipocytes, is a pivotal feature of MSCs. We showed that 3D MSC spheroids maintained their differentiation capabilities after being subjected to mechanical stress induced by cell application through a microcatheter. In line with our results, Lee et al. showed that injectable 3D spheroids of MSC improve the engraftment efficiency after transplantation [52]. Moreover, after the implantation of spheroids, MSC are capable to undergo differentiation processes into suitable cells for reconstructing the diseased tissue [53,54].

The behavior and trackability of USPIO labelled spheroids injected in intact peripheral intramuscular vessels was investigated in an ex vivo hind limb rabbit model via MRI imaging. USPIO labelled MSC spheroids were easily distinguishable throughout the entire vascular territory and had a homogenous distribution in the targeted arteries. Our studies are in accordance with previous findings where labelling of MSCs with USPIO proved to be a nontoxic and feasible procedure in a clinical context, the cells being easily detectable via MRI [37,39,55]. Post application effects of applied MSC spheroids have also been investigated. Bhang et al. showed that transplantation of MSCs spheroids in a mouse ischemic hind limb yielded an overall improved survival of translated cells, increasing the number of de novo microvessels and smooth muscle α-actin-positive vessels attenuating limb loss and necrosis [36]. Similarly, in an in vivo rat skin repair model, tracking of fluorescently labelled MSCs indicated a precise localization of MSC spheroids in micro vessels, suggesting enhanced angiogenesis effects [56]. Histologic evaluation revealed plugging of vessels with diameters of 500 µm, while the mean size of the initial applied spheroids measured 1200 µm. This emphasizes the ability of spheroids to deform and adapt to the emerging microenvironment settings when exposed to mechanical stress while maintaining their integrity.

It is conceivable that cell spheroids can be applied to the environment of a diseased vasculature via an endovascular access like intra-arterial catheters during an infragenicular interventional procedure. Spheroids are feasible candidates for clinical translation, as donors isolated cells can be cultured with the donor’s own plasma under GCP regulations and conditions and afterwards transplanted back at the site of injury. In such any adverse immune reactions are prevented. The number of cells applied in human studies ranges from 10^6^ to 10^9^ cells injected into calf muscles or into the peripheral artery [57,58]. A possible advantage of spheroid’s application is that smaller initial cell densities are required due to the higher survival rate in the targeted region after application. Overall, our study showed that USPIO labelling of MSC spheroids and application under variable conditions does not impair the cellular viability, outgrowth, differentiation potential and trackability as assessed by MRI. In further research efforts, the labelling can be systemically employed for cell tracking in in vivo models, thus revealing insights about the exact behaviour under blood flow conditions and the migration capacities of the MSC.

## 4. Materials and Methods

### 4.1. Production and Size Distribution of 3D Cell Spheroids

Human bone marrow derived MSC were isolated at the University Hospital Tübingen after written informed consent of the patients. Full approval has been obtained from the local ethics committee (approval number: 401/2013BO2, date: 27 August 2013). Cells were isolated as previously described [59]. All cells were cultivated in Alpha-MEM (Gibco/Thermofisher, Waltham, MA, USA) + 10% (*v*/*v*) fetal calf serum (FCS, Lonza Group AG, Basel, Switzerland) +1% (*v*/*v*) Penicillin Streptomycin at 37 °C in a humidified atmosphere containing 5% CO_2_. For 3D MSC spheroids production, cells on passage 3–5 were used. After reaching 80% confluence, cells were detached and counted with a cell counter (CASY Model TT Cell Counter and Analyzer, Roche Diagnostics GmbH, Mannheim, Germany).

For generation of 3D spheroids, MSC were seeded into low-attachment 96- well plates (Thermo Nunclon Sphera Plates, Thermo Scientific, Waltham, MA, USA) at densities of 125,000, 62,500 or 31,250 cells /well, as the variability was expected to increase with higher initial numbers following the manufacturer’s instructions. After seeding and incubating the cells for 5 days under standard cell culture conditions (37 °C, 5% CO_2_, Normoxia) cells grew in a 3D shape, forming stable spheroids (Figure 1) [37].

After 5 days in culture, the diameters of the MSC spheroids were assessed by means of an inverse light microscope (DM 100, Leica, Wetzlar, Germany) and photographed (Canon EOS 350D, Canon, Krefeld, Germany). The diameter of unlabelled cell spheroids was assessed with Image J software (National Institutes of Health, Bethesda, MD, USA). Grid lines of a Neubauer Improved Cell Counting Chamber (Hecht, Sondheim Rhön, Germany) served as a size standard.

### 4.2. Scanning Electron Microscopy

Scanning electron microscopy of both labelled, and unlabelled cell spheroids was performed with a Hitachi TM1000 (Hitachi, Tokio, Japan). Prior to imaging, cell spheroids were washed with phosphate buffered saline (PBS, Sigma-Aldrich, Taufkirchen, Germany) and fixed with a 4% (*w*/*v*) paraformaldehyde in PBS at 4 °C overnight. After further washing steps with PBS, cell spheroids were dehydrated in a series of ethanol solutions with increasing concentration (30% to 100%). For analysis of the spheroids morphology different objective magnifications were employed (from 200× to 1000×).

### 4.3. Injection Experiments: Mechanical Stability and Viability

To evaluate the mechanical stability, viability and functionality of the spheroids after passage through different catheter sizes, we developed a capillary model to simulate the passage through an application catheter. The inner diameters of the flexible tubes were 1050 µm, 690 µm, 530 µm and 400 µm, with a length of 100 cm. Routinely, the spheroids were applied through the tubes with a velocity of 90 µL/s and collected in well plates containing Alpha-MEM + 10% (*v*/*v*) fetal calf serum +1% (*v*/*v*) penicillin streptomycin for further analyses. To assess the impact of different flow rates, a microcatheter (Progreat Micro Catheter, Shibuya, Japan) was used with an inner diameter of 530 µm and a length of 130 cm, and spheroids were applicated with flow rates of 90 µL/s (referred to as ‘low’) or 200 µL/s (referred to as ‘high’). For determination of viability, collected spheroids were transferred into new well plates to minimize background effects caused by abraded cells and cellular debris. Disrupted but cohesive spheroids were utilized for viability tests (Figure 3C) but not for size determination (Figure 3B). Completely fragmented spheroids (i.e., ones obtained from applied 125,000 cell spheroids) were not used neither for size determination nor viability assessment.

The diameter of the applied spheroids was quantified by microscopic photography with 2.5x magnification and by subsequent usage of ImageJ software (National Institutes of Health, Bethesda, MD, USA).

#### Viability Assay

The metabolic and proliferative potential of pre-treated (tubes/microcatheter) or non-treated spheroids was measured using 3-(4,5-dimethylthiazol-2-yl)-5-(3 carboxymethoxyphenyl)-2-(4-sulfophenyl)-2H-tetrazo-lium (MTS) assay (Promega CellTiter 96^®^ Aqueous One Solution, Madison, WI, USA) following the manufacturer’s instructions. In short, Spheroids were collected immediately after application and seeded into 48-wells containing culture medium. MTS solution was added in a ratio of 1:6, and the spheroids were cultured for 16 h under standard conditions (37 °C, 5% CO_2_). The absorbance of the culture supernatants was measured at a wavelength of 490 nm with an EL800 microplate reader (BioTek Instruments GmbH, Bad Friedrichshall, Germany) and normalized against medium controls. Results are presented as signals per single spheroids.

### 4.4. Differentiation Potential of MSCs after Passage through Different Lumen Sizes

The differential potential of applicated and of non-delivered spheroids was assessed. For this purpose, spheroids from both settings were cultured initially in standard culture medium for 3 to 4 weeks until sufficient outgrow of cells occurred. Subsequently, osteogenic, adipogenic or chondrogenic differentiation was induced by cultivation in the respective induction medium according to the manufacturer’s instructions (Lonza Group AG, Basel, Switzerland) and cells were stained as described below. Several spheroids from both settings were held in normal culture medium (see above), representing non-differentiated negative controls.

To evaluate the osteogenic capacity, the spheroids were cultured in hMSC osteogenic induction medium (Lonza) for 3 to 4 weeks with media changes twice a week. The induction medium was compiled from the hMSC osteogenic BulletKit^®^ (Lonza PT-3002). After paraformaldehyde fixation of the cells, calcium deposits of osteogenic cultures were stained with 0.5% (*w*/*v*) Alizarin Red S dye (Sigma-Aldrich, Taufkirchen, Germany), pH 4.1 for 20 min.

Adipogenic differentiation was induced by alternate incubation of cells in hMSC adipogenic induction medium and in hMSC adipogenic maintenance medium (both from Lonza) for 3 to 4 days each. Media were compiled from the hMSC adipogenic BulletKit^®^ (Lonza PT-3004). After 3 cycles of induction and maintenance, cells were fixed with 4% paraformaldehyde for 30 min, incubated in 60% isopropanol for 5 min and stained with 0.24% (*w*/*v*) Oil Red O (Sigma-Aldrich, Taufkirchen, Germany) in 60% isopropanol for 20 min to detect lipid vacuoles.

To induce chondrogenic differentiation, spheroids were first cultured for 3 to 4 weeks in standard culture medium until outgrowing MSC reached a confluence of 100%. Medium was replaced by hMSC chondrogenic differentiation medium (Lonza) for another 3 to 4 weeks with medium changes twice a week. The differentiation medium was compiled from the hMSC chondrogenic BulletKit^®^ (Lonza PT-3003) and was additionally supplemented with 10 ng/mL hTGF-beta3 (Miltenyi Biotec, Bergisch Gladbach, Germany). Finally, cells were fixed with 4% paraformaldehyde overnight at 4 °C, and proteoglycans were stained with 1% (*w*/*v*) Alcian Blue 8-GX (Sigma-Aldrich, Taufkirchen, Germany) in 3% acetic acid for 15 min at room temperature.

### 4.5. MR-Imaging

#### 4.5.1. USPIO—Labelling

Cells were cultured in a 2D monolayer until sub confluence (max. 80%) under standard cell culture conditions (37 °C, 5% CO_2_, Normoxia). Subsequently the cells were cultivated in culture media with 60 µg/mL Ferucarbotran (Meito Sangyo Co. Ltd., Nagoya, Japan) for 24 h, as previously described [60]. Briefly, the cells were detached and counted by an automated cell counter (CASY Model TT Cell Counter and Analyzer, Roche Diagnostics GmbH, Bremen, Germany). Unlabelled cells served as a control.

Ferucarbotran (Meito Sangyo Co. Ltd., Nagoya, Japan) previously distributed solely under the brand name Resovist^®^ within BAYER Schering AG (Berlin, Germany) is a clinically approved superparamagnetic iron oxide developed for contrast-enhanced MRI. MSC cells were cultured in media containing Ferucarbotran for MRI-trackability.

#### 4.5.2. Agar Phantom Measurements

An agar matrix was used as appropriate environment for measuring the iron load of the USPIO-labelled cell spheroids. In detail, an agar solution 1% (*w*/*v*) (Sigma-Aldrich, Taufkirchen, Germany) in PBS was boiled and gradually cooled down in a water bath. This slow cooling procedure reduced the inclusion of air bubbles into the matrix, in order to minimize the occurrence of artefacts during measurement. Before getting stable, the agar matrix was embedded in plastic boxes. By utilization of a special stamp, a series of cone shaped cavities was formed in the agar block. For MRI measurements, labelled and unlabelled MSC 3D spheroids were placed in the formed cavities and embedded with 4% (*w*/*v*) gelatine (Sigma-Aldrich, Taufkirchen, Germany) in PBS. A cellular density of 125,000 MSCs was used for the experiments. In the Agar phantom a dilution series of Ferucarbotran with 28, 14, 7, 3.5, 1.8, 0.9, 0.45, 0.22 µg was employed. MRI measurements were performed on a whole body 3T MR scanner (MAGNETOM PrismaFit, Siemens Healthcare, Erlangen, Germany). A single-slice multi-echo spoiled gradient-echo sequence was used. Sequence parameters were as follows: number of echoes, 12; 1st echo time, 2.65 ms; echo time increment, 4.06 ms; repetition time, 1000 ms; flip angle, 25°; slice thickness, 2 mm, in-plane resolution, 0.42 × 0.42 mm^2^, and readout bandwidth, 645 Hz/pixel.

#### 4.5.3. Ex Vivo Injection of 3D Spheroids in Hind Limb Rabbit Arteries

To investigate the distribution of the 3D cell spheroids in normal vessel anatomy, an injection into freshly harvested cadaveric rabbit hind limbs (*n* = 3) was conducted. Cells were prepared and labelled as beforehand described (Section 4.5.1 USPIO labelling). The femoral artery was exposed and a 19G injection needle was inserted. The vessel was then ligated with surgical sutures. A PBS solution containing the USPIO labelled spheroids (30 USPIO labelled—spheroids) was carefully injected. To evaluate the distribution and the disposition in the arterial vessels, an MRI scan was conducted directly after the procedure. Imaging was conducted on a 3T scanner (Prisma fit, Siemens, Erlangen, Germany) using T2 weighted gradient sequences.

### 4.6. Ex-Vivo Application/Pathologic Examination

The aim of the ex vivo application was to assess the spheroid delivery success in the targeted vessel region. After MRI measurements the tissue was harvested and fixed in a 4% (*w*/*v*) PFA solution in PBS for 30 min at room temperature. Conventional sections 15 µm in thickness were cut with a Leica cryotome type CM3050S (Leica Biosystems, Wetzlar, Germany). The sections were observed under 25-fold microscopic magnification, light microscope (DM 100, Leica, Wetzlar, Germany).

### 4.7. Statistical Analysis

Data is presented as box and whisker plots or as bar charts showing mean values ± standard error of the mean, respectively. The differences between groups were assessed by means of a two-tailed Mann-Whitney test, using GraphPad Prism version 8.4.0 (GraphPad Software Inc., San Diego, CA, USA). Values of * *p* < 0.05 or ** *p* < 0.01 were considered to be statistically significant.

## 5. Conclusions

Our study showed that injection of MSC spheroids through catheters with various lumen sizes did not impair the cellular viability, outgrowth and differentiation potential. USPIO labelled MSC spheroids were easily identified by MRI in an ex vivo rabbit hind limb model. The spheroids were retained in the microvasculature and their distribution were precisely visualized. Overall, MSC spheroids represent a reliable method for intravascular application in the context of ischemic peripheral vascular disease. Future research should investigate the migration and integration of cells into the surrounding tissue, their ability to form de novo micro vessels and regenerative effects.

## Data Availability

Data is available to colleagues upon reasonable request.
